# First report of *Rickettsia felis*in China

**DOI:** 10.1186/s12879-014-0682-1

**Published:** 2014-12-16

**Authors:** Jilei Zhang, Guangwu Lu, Patrick Kelly, Zhenwen Zhang, Lanjing Wei, Duonan Yu, Shayilan Kayizha, Chengming Wang

**Affiliations:** Jiangsu Co-innovation Center for the Prevention and Control of Important Animal Infectious Diseases and Zoonoses, Yangzhou University College of Veterinary Medicine, Yangzhou, Jiangsu China; Ross University School of Veterinary Medicine, Basseterre, Saint Kitts and Nevis; Subei People’s Hospital, Yangzhou, Jiangsu China; Yangzhou University School of Medicine, Jiangsu, China; Institute of Veterinary Science, Xinjiang Academy of Animal Science, Urumqi, 830000 China

**Keywords:** Rickettsia felis, China, Serology, PCR

## Abstract

**Background:**

*Rickettsia felis* is a recently described flea-borne spotted fever group *Rickettsia* that is an emerging human pathogen. Although there is information on the organism from around the world, there is no information on the organism in China.

**Methods:**

We used a commercial ELISA to detect antibodies reactive against *R. felis* in blood samples and developed a PCR to detect the *gltA* of the organism in blood samples and external parasites.

**Results:**

We found reactive antibodies in people (16%; 28/180), dogs (47%; 128/271) and cats (21%; 19/90) and positive PCRs with DNA from people (0.1%; 1/822), dogs (0.8%; 8/1,059), mice (10%; 1/10), ticks (*Rhipicephalus sanguineus*; 10%; 15/146), lice (*Linognathus setosus*; 16%; 6/37), fleas (*Ctenocephalides felis felis*; 95%; 57/60) and mosquitoes (*Anopheles sinensis, Culex pipiens pallens*; 6%; 25/428), but not from cats (0/135) or canine fecal swabs (0/43).

**Conclusions:**

This is the first report of *R. felis* in China where there is serological and/ or PCR evidence of the organism in previously reported [people, dogs, cats, ticks (*Rhipicephalus sanguineus*), fleas (*Ctenocephalides felis felis*) and mosquitoes (*Anopheles sinensis, Culex pipiens pallens*)] and novel species [mice and lice (*Linognathus setosus*)].

**Electronic supplementary material:**

The online version of this article (doi:10.1186/s12879-014-0682-1) contains supplementary material, which is available to authorized users.

## Background

Although tick-borne spotted fever group rickettsiae have been described in China [1], there is no information on the flea-borne emerging human pathogen *Rickettsia felis*. Described in 2001, *R. felis* appears to have the cat flea, *Ctenocephalides felis felis*, as its main vector and reservoir and can infect other arthropods (mosquitoes, ticks and mites) and mammals (rats, opossums, dogs, and cats). It is found worldwide and, in Asia, it has been definitively identified by molecular methods in fleas (Indonesia, Thailand, Afghanistan, South Korea, Laos, Malaysia, Taiwan), ticks (Japan), raccoons (Japan) and people (Taiwan, South Korea) [2],[3]. To expand our knowledge on *R. felis* in Asia, we studied people, animals and arthropods from around China using serology and molecular techniques.

## Methods

### Samples collection

**Figure 1:**
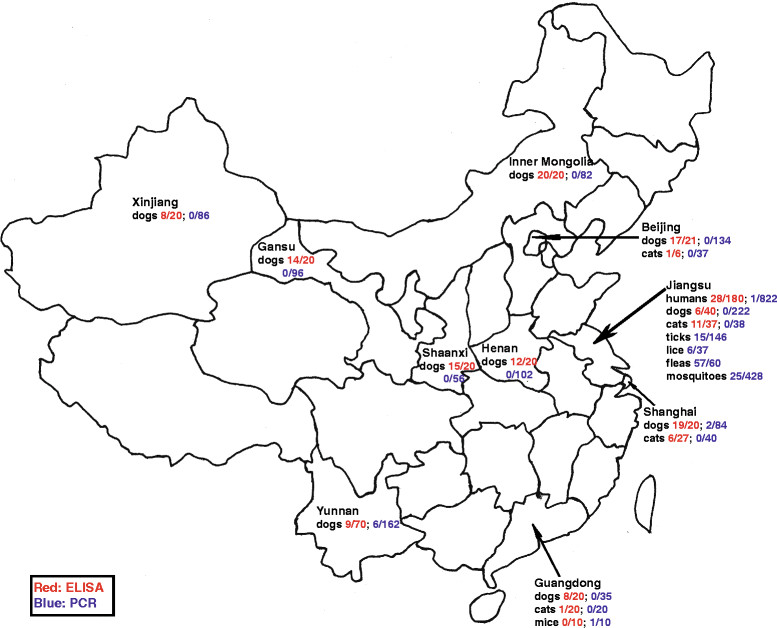
**Sites in China where samples were obtained for** ***R. felis*** **testing by ELISA and PCR.** People sampled in Jiangsu province were apparently healthy individuals attending the Subei People’s Hospital for routine health checks. Dogs sampled in Taixing of Jiangsu were apparently healthy animals in a breeding kennel and parts of those from Gansu province were from a shelter while all other dog samples were obtained from patients with a variety of conditions attending the veterinary clinic. The cats sampled from Jiangsu were apparently healthy animals in a shelter while those from Beijing, Shanghai and Guangdong were from the veterinary clinic with variety conditions. In Jiangsu, ticks and lice were obtained from breeding kennel dogs and fleas were obtained from feral cats. The mice were captured in traps in Guangdong and the mosquitoes were captured with hand-nets in the environs of the Yangzhou University of Jiangsu.

This study was approved by the Institutional Animal Care and Use Committee of Yangzhou University and the Institutional Review Board of Subei People’s Hospital, China. Written permission was obtained from participants and owners of animals that participated in the study. People sampled in Jiangsu province (Figure 1) were apparently healthy individuals attending the Subei People’s Hospital for routine health checks. Dogs sampled in Taixing of Jiangsu were apparently healthy animals in a breeding kennel while those from Gansu province were from a shelter. All other dog samples were obtained from patients with a variety of conditions attending local veterinary clinics. The cats sampled in Jiangsu were apparently healthy animals in a shelter while those from Beijing, Shanghai and Guangdong were from animals presenting to veterinary clinics with a variety of conditions. In Jiangsu, ticks and lice were obtained from breeding kennel dogs and fleas were obtained from feral cats. Mice and shrews were captured in traps in Guangdong and the mosquitoes were captured with hand-nets in the environs of the Yangzhou University of Jiangsu.

Plasma and buffy coats from people, dogs, cats and wild mice (Figure 1) were stored at −80°C until DNA extraction. Rectal swabs from dogs and organs (spleen, liver and kidney) from the humanely euthanized wild mice were stored at −80°C in 800 μL of RNA/DNA Stabilization Reagent for Blood/Bone Marrow (Roche Molecular Biochemicals, Indianapolis) until DNA extraction. The external parasites collected from dogs and cats, and mosquitoes (Figure 1) were identified using standard morphological criteria and stored as above.

### Serology assay

The *R. felis* EIA IgG Antibody Kit (Fuller Laboratory, USA) was used according to the manufacturer’s instructions with peroxidase-conjugated AffiniPure Goat Anti-Cat, Rabbit Anti-Dog, and Goat Anti-Mouse IgG (H + L) (Jackson ImmunoResearch Laboratories, USA) substituted as secondary antibodies for cat, dog and mouse/shrew assays, respectively. For human plasma, the cut-off level was determined following the manufacturer’s instructions that an index (OD value of test serum divided by the average OD values of the Cutoff Calibrator) above 1.2 should be considered positive. Plasma from cats, mice, shrews and dogs was regarded as positive if they gave an OD value above the mean plus three standard deviations of the respective negative control samples [4],[5].

### DNA extraction

Samples were thawed at room temperature and DNA was extracted from buffy coats, homogenized organs and arthropods [6], and canine rectal swabs with the QIAamp® DNA Blood Mini Kit (QIAgen, Valencia, USA), QIAamp® DNA Mini Kit, and QIAamp® DNA Stool Mini Kit, respectively, following the manufacturer’s protocol.

### PCR assays

**Figure 2:**
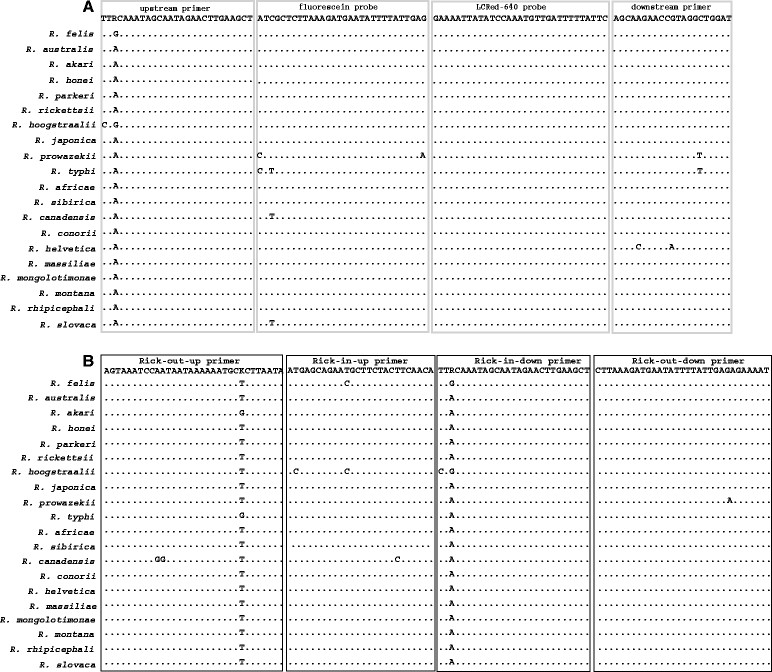
**Alignment of the primers and probes for the** ***gltA*** **-based FRET-qPCR and nested PCR with 20** ***Rickettsia*** **species.** Panel **A** shows the nucleotide sequences of the primers and probes used in the FRET-qPCR and the corresponding sequences of 20 *Rickettsia* species. Panel **B** shows the nucleotide sequences of the primers used for the nested PCR. In both Panels, dots indicate that nucleotides are identical to the primers. The nucleotides between oligonucleotides are not shown. The upstream primer and probes were used as shown while the downstream primer was used as an antisense oligonucleotide.

Using the Clustal Multiple Alignment Algorithm we identified a conserved region of the *gltA* in 20 representative *Rickettsia* species. Primers and probes were designed to amplify a 170-bp target using a FRET-PCR, and 446-bp and 353-bp targets using a nested-PCR (Figure 2). The PCRs were performed in a LightCycler® 480II PCR platform with hydroxymethylbilane synthase as an endogenous internal control [6]. Ten microliters of extracted DNA was tested in a 20 μL final volume of reaction mixture. Thermal cycling consisted of a denaturation step (2 min @ 95°C) and 18 high-stringency step-down cycles followed by 40 relaxed-stringency fluorescence acquisition cycles. The 18 high-stringency step-down thermal cycles were 6 × 1 sec @ 95°C, 12 sec @ 70°C, 8 sec @ 72°C; 9 × 1 sec @ 95°C, 12 sec @ 68°C, 8 sec @ 72°C; 3 × 1 sec @ 95°C, 12 sec @ 66°C, 8 sec @ 72°C. The relaxed-stringency fluorescence acquisition cycling consisted of 40 × 1 sec @ 95°C, followed by fluorescence acquisition of 8 sec @ 57°C, and 30 sec @ 72°C. Melting curve analysis for probes annealing to the PCR products was performed by monitoring the fluorescence from 38°C to 85°C with the first derivatives of F4/F1 being evaluated to determine the probe melting temperature (*T*_m_). For nested-PCR, the PCR steps were the same as those in the FRET-PCR with the exclusion of the melting step. Positivity of samples was confirmed using gel electrophoresis with the SYBR® safe DNA Gel Stain (Invitrogen™, Carlsbad, CA, USA) and genomic sequencing conducted by a commercial company (GenScript, Nanjing, China). Two ultramer oligos (Integrated DNA Technologies, USA) containing portions of the *gltA* of *R. felis* and *R. typhi* were used to prepare quantitative standards (10^4^ to 10^0^*gltA* copies/10 μL) and establish the sensitivity. All the PCR assays were performed with plasmid standards and sterile H2O as positive and negative controls, respectively.

## Results and discussion

The ELISAs showed high prevalences of antibodies to *R. felis* in people (16%; 28/180), dogs (47%; 128/271) and cats (21%; 19/90) (Figure 1, Table 1). Previous serosurveys have shown similar numbers of apparently healthy people in Colombia (18%), Spain (7%), Senegal (4%), and Kenya (3%) [7],[8] are seropositive. There were no differences in age or complete blood count (CBC) parameters between sero-positive and sero-negative people. People that were ELISA positive had an average age of 45.03 years ±14.57 and their complete blood count parameters were RBC: 4.76 × 10^12^/L ± 0.42, HCT: 43.52% ±3.57, WBC: 6.20 × 10^9^/L ±1.73, NE%: 58.61 ± 8.35, and PLT: 211.00 × 10^9^/L ±48.20. The average age of the sero-negative people was 45.15 years ±14.61 and their blood parameters were RBC: 4.76 × 10^12^/L ±0.42, HCT: 43.55% ±3.59, WBC: 6.20 × 10^9^/L ±1.74, NE%: 58.54 ± 8.41, and PLT: 211.03 × 10^9^/L ±47.66.

**Table 1:** **Details of samples collected in China and the results of ELISA and PCR testing**

Sample type	Source of samples			Sample number	Sero-positivity (%), pos/total samples	PCR-positivity (%), pos/total samples
	Province/ unicipality	City	Coordinates			
Human blood	Jiangsu	Yangzhou	32°N, 119°E	822	15.6%, 28/180	0.1%, 1/822
Dog blood	Beijing	Beijing	39°N, 116°E	134	81.0%, 17/21	0.0%, 0/134
	Gansu	Lanzhou	36°N, 103°E	96	70.0%, 14/20	0.0%, 0/96
	Guangdong	Guangzhou	23°N, 113°E	35	40.0%, 8/20	0.0%, 0/35
	Henan	Zhengzhou	34°N, 113°E	102	60.0%, 12/20	0.0%, 0/102
	Inner Mongolia	Huhhot	40°N, 111°E	82	100.0%, 20/20	0.0%, 0/82
	Jiangsu	Yangzhou	32°N, 119°E	50	25.0%, 5/20	0.0%, 0/50
		Taizhou	32°N, 120°E	111	0.0%, 0/10	0.0%, 0/111
		Nanjing	32°N, 118°E	61	10.0%, 1/10	0.0%, 0/61
	Shanghai	Shanghai	31°N, 121°E	84	95.0%, 19/20	2.4%, 2/84
	Shaanxi	Yangling	34°N, 108°E	56	75.0%, 15/20	0.0%, 0/56
	Xinjiang	Urumchi	43°N, 87°E	86	40.0%, 8/20	0.0%, 0/86
	Yunnan	Kunming	25°N, 102°E	162	12.9%, 9/70	5.8%, 6/162
Cat blood	Beijing	Beijing	39°N, 116°E	37	16.7%, 1/6	0.0%, 0/37
	Guangdong	Guangzhou	23°N, 113°E	20	5.0%, 1/20	0.0%, 0/20
	Jiangsu	Yangzhou	32°N, 119°E	38	29.7%, 11/37	0.0%, 0/38
	Shanghai	Shanghai	31°N, 121°E	50	22.2%, 6/27	0.0%, 0/50
Lice	Jiangsu	Taizhou	32°N, 120°E	37	NA*	16.2%, 6/37
Tick	Jiangsu	Taizhou	32°N, 120°E	146	NA	10.3%, 15/146
Cat flea	Jiangsu	Yangzhou	32°N, 119°E	60	NA	95.0%, 57/60
Mosquito	Jiangsu	Yangzhou	32°N, 119°E	664	NA	6.3%, 42/664
Dog Rectal swab	Yunnan	Kunming	25°N, 102°E	43	NA	0.0%, 0/43
Mouse blood	Guangdong	Zhanjiang	21°N, 110°E	10	NA	0.0%, 0/10
Liver				10	NA	0.0%, 0/10
Kidney				10	NA	0.0%, 0/10
Spleen				10	NA	10.0%, 1/10

Seropositive dogs were found in each area studied with prevalences from 13-100%, similar to the 51% reported in Spain and Australia, and ≤13% in Brazil [9],[10]. There were significantly fewer seropositive cats (5-30%; two-tailed chi square analysis, *P* < 10^−4^), similar to the low level reported in the US (< 11%) [11]. All mice (6 *Mus musculus*) and shrews (4 *Suncus murinus*) that were trapped were seronegative.

Although it has been suggested there might be high specificity of *R. felis* in serological tests [9] there is at least some serological cross reactivity between *R. felis* and other *Rickettsia* spp. present in China [1]. We therefore used PCR to definitively identify *R. felis* in our study populations and provide further prevalence data. The GenBank BLAST program showed primers and probes of our FRET-PCR and nested PCR recognized all *Rickettsia* spp., but none in the other genera of the Rickettsiaceae. The detection limit of our combination PCR system was one gene copy per 20 μl reaction system.

Our PCRs showed one person had DNA of *R. felis* (0.1%; 1/822), a twenty-seven year old man with a normal CBC who was seronegative. This might have been an acute infection or an asymptomatic infection with no serological response as reported previously [8]. Dogs from 2 of the 10 areas studied were also PCR positive (0.8%; 8/1,059), similar to Australia where up to 9% of dogs are PCR positive [9]. Previously, *R. felis* was found in feces from great apes in Africa [12] but all our canine rectal swabs were negative by PCR. These dogs, however, had negative serology and blood PCRs and were probably then not infected.

As found previously [13], all the cats we studied were negative by PCR, despite many being seropositive and many harboring PCR positive *Ctenocephalides felis*, cat fleas. Fleas were the only ectoparasites found on cats and almost all were PCR positive (95%; 57/60), consistent with the very high levels of infection found worldwide and the generally accepted hypothesis that cat fleas are the primary arthropod vectors and reservoirs [3]. Our finding that dogs have a higher seroprevalence and are positive by PCRs supports the hypothesis that they, rather than cats, might be the main mammalian reservoir of *R. felis* [14].

The spleen of one *M. musculus* was PCR positive for *R. felis* which is the first definitive report of the organism in mice. A PCR positive *Rattus norvegicus* has also been reported [15] and investigation into the role of rodents in the epidemiology of *R. felis* appears warranted.

All the amplicons we obtained in the above PCRs had identical sequences to those of the R. felis type strain URRWXCal2 (CP000053). In addition the amplicons had identical sequences with other strains of *R. felis* including those with GenBank sequences JQ674484 (from *Aedes albopictus* mosquitoes, Libreville, Gabon) and JN375498 (from *Canis familiaris* in Southeastern Brazil). We submitted sequences obtained from two dogs to GenBank along with some of the sequences described below (Table 2).

**Table 2:** **Details of the sequences we obtained in our study and submitted to GenBank**

GenBank No.	Submission No.	Species
KJ440515	1698905	Dog - *Canis lupus familiaris*
KJ440516	1698976	Dog - *Canis lupus familiaris*
KJ440517	1698981	Louse - *Linognathus setosus*
KJ440518	1698985	Louse - *Linognathus setosus*
KJ440519	1698989	Mosquito - *Anopheles sinensis*
KJ440520	1699024	Mosquito - *Culex pipiens pallens*
KJ440521	1699027	Tick - *Rhipicephalus sanguineus*
KJ440522	1699029	Louse - *Linognathus setosus*
KJ440523	1699030	Mosquito - *Anopheles sinensis*

Ten percent of the ticks (146 *Rhipicephalus sanguineus*) we collected from dogs were PCR positive. The sequences were all identical to *R. felis* (CP000053) except one (KJ440521) which was 99% identical to *R. felis* (CP000053) and *R. typhi* (U59714). Sixteen percent (6/37) of the dog lice (all *Linognathus setosus*) were positive; four had amplicons identical to *R. felis* (CP000053) while two amplicons from dog lice (KJ440522) were similar to *R. endosymbiont* (EU760765) (97%), *R. bellii* (U59716) (96%) and *R. felis* (CP000053) (86%). While *R. felis* has been reported in *R. sanguineus* in South America [16], ours is the first report of the organism in lice. All the dogs with PCR positive lice or ticks were sero- and PCR negative for *R. felis* suggesting these arthropods might not be competent vectors.

Six percent (25/428) of the mosquitoes (32 *Anopheles sinensis*, 396 *Culex pipiens pallens*) were PCR positive with 23 (2 *An. sinensis*, 21 *C. p. pallens*) having sequences identical to *R. felis* (CP000053) and 2 (*C. p. pallens*) having 99% and 96% similarity. The latter was 99% identical to a novel *Rickettsia* sp. (JN620082) found in *An. gambiae* in Africa which may be a new human pathogen [17]. Ours is the first report of *R. felis* and the new *Rickettsia* sp. in mosquitoes outside of Africa and the first of the organisms in *An. sinensis* and *C. p. pallens*. Further studies are indicated to determine the role of mosquitoes in the epidemiology of these rickettsias and the influence of the *Rickettsia* spp. might have on the biology of mosquitoes.

## Conclusions

Our study indicates that *R. felis* occurs widely in China and infects a variety of previously reported (people, dogs, cats, ticks, fleas and mosquitoes) and novel species (mice and lice). Further studies are indicated to investigate the epidemiology and transmission mechanisms of *R. felis*, particularly in mosquitoes, lice and mice.

## Authors’ original submitted files for images

Below are the links to the authors’ original submitted files for images. Authors’ original file for figure 1 Authors’ original file for figure 2
