# Supplementary figures and images for: First report of Rickettsia felisin China
Source: BMC Infect Dis. 2014 Dec 16;14:682. doi: 10.1186/s12879-014-0682-1 (PMC4297373; doi:10.1186/s12879-014-0682-1)

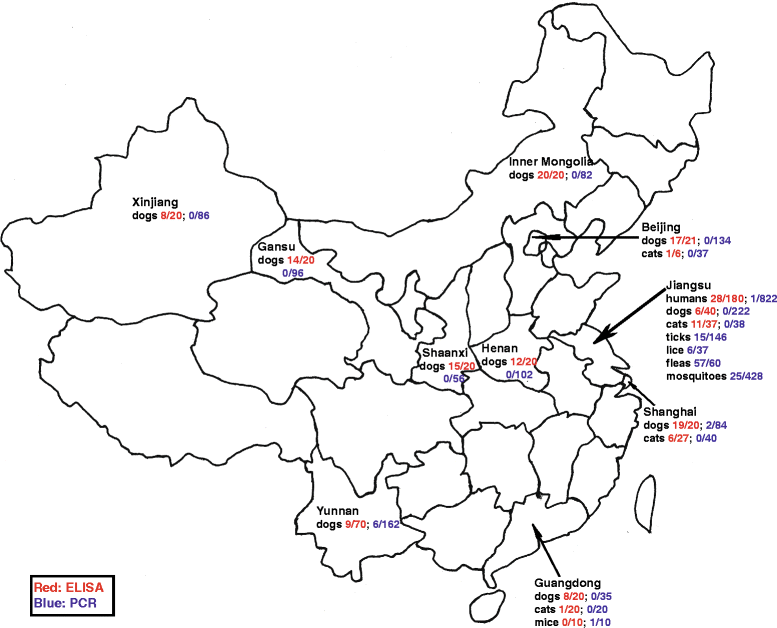

Supplement: Supplementary file 1 — Authors’ original file for figure 1 [file 12879_2014_682_MOESM1_ESM.gif]

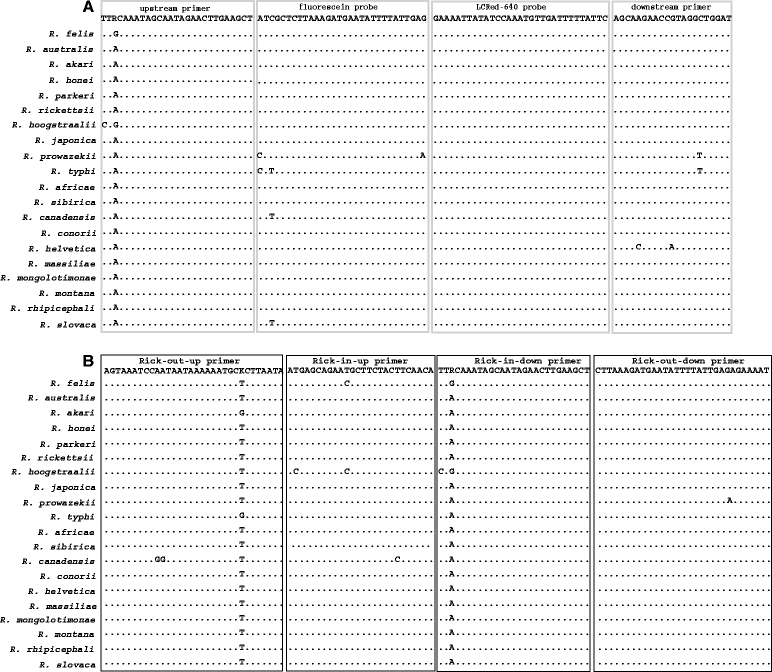

Supplement: Supplementary file 2 — Authors’ original file for figure 2 [file 12879_2014_682_MOESM2_ESM.gif]
